# Experimental pathology by intravital microscopy and genetically encoded fluorescent biosensors

**DOI:** 10.1111/pin.12925

**Published:** 2020-04-08

**Authors:** Michiyuki Matsuda, Kenta Terai

**Affiliations:** ^1^ Department of Pathology and Biology of Diseases, Graduate School of Medicine Kyoto University Kyoto Japan; ^2^ Research Center for Dynamic Living Systems, Graduate School of Biostudies Kyoto University Kyoto Japan

**Keywords:** biosensor, experimental pathology, FRET, intravital microscopy

## Abstract

The invention of two‐photon excitation microscopes widens the potential application of intravital microscopy (IVM) to the broad field of experimental pathology. Moreover, the recent development of fluorescent protein‐based, genetically encoded biosensors provides an ideal tool to visualize the cell function in live animals. We start from a brief review of IVM with two‐photon excitation microscopes and genetically encoded biosensors based on the principle of Förster resonance energy transfer (FRET). Then, we describe how IVM using biosensors has revealed the pathogenesis of several disease models.

**Abbreviations**
Cdk1: cyclin‐dependent kinase 1
CFP: cyan fluorescent protein
EGF: epidermal growth factor
EGFR: epidermal growth factor receptor
ERK: extracellular signal‐regulated kinase
EPR: enhanced permeability and retention
FRET: Förster resonance energy transfer
H&E: hematoxylin‐eosin
HUVECs: human umbilical vascular endothelial cells
IVM: intravital microscopy
MMTV: mouse mammary tumor virus
PKA: protein kinase A
VEGF: vascular endothelial growth factor
YFP: yellow fluorescent protein
2P: two‐photon
2PEM: 2P excitation microscopy

## INTRODUCTION

The field of modern cellular pathology began with the introduction of light microscopy and hematoxylin‐eosin (H&E) staining in the late 19th century, and reached a zenith in the early 20th century, when a number of academic feats were achieved by pathologists. Even if we restricted ourselves to the history of the Japan Society of Pathology, we can easily recall the chemically induced squamous cell carcinoma in rabbits by Katsusaburo Yamagiwa and Kōichi Ichikawa,^1^ and the discovery of chicken sarcoma virus by Akira Fujinami,^2^ which heralded the era of chemical carcinogenesis and viral oncology, respectively. Thereafter, experimental pathology has continued to evolve by adopting cutting‐edge technologies in each successive period, such as electron microscopy, immunohistochemistry, *in‐situ* hybridization and so on. However, such techniques can be applied only to a fixed sample. Researchers in experimental pathology have long desired to microscopically observe the tissues in live experimental animals. Moreover, the field of experimental pathology was somewhat neglected during the flourishing of biochemistry and molecular biology in the late 20th century, because these fields tend to handle tissue samples as a mass for the analysis of molecular activities rather than considering the heterogeneity at a cellular resolution in the manner of pathologists. Now, the goal of observing the molecular activities in the diseased tissue of live animals has been realized through the development of intravital microscopy (IVM) with a two‐photon (2P) microscope and various fluorescent probes. Here, we summarize the uses of IVM with a 2P microscope in experimental pathology, with a particular focus on the use of genetically encoded fluorescent biosensors.

## TWO‐PHOTON EXCITATION MICROSCOPY AND GENETICALLY ENCODED FLUORESCENCE BIOSENSORS

### Two‐photon excitation microscopy

**Figure 1:**
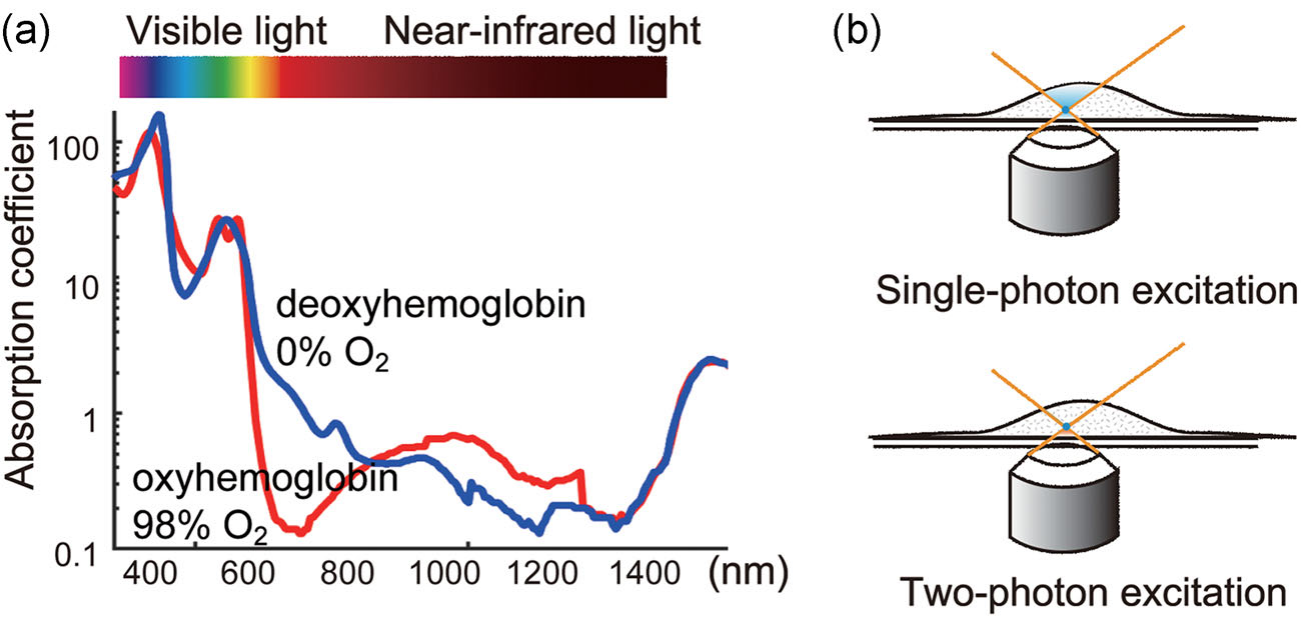
Properties of two‐photon excitation microscopy (2PEM) using a near‐infrared high power femtosecond (fs) pulsed laser. (**a**) The light absorption of hemoglobin is the lowest, in the wavelength range of 700 to 1300 nm, which is often referred to as the near‐infrared window in biological tissue. The spectrum is from Bosschaart *et al*.^5^ (**b**) In the conventional single‐photon fluorescence microscopy, all fluorescence molecules along the light path are excited. In 2PEM, two photons must reach the single fluorophore almost simultaneously, which occurs only at the focal plane, markedly reducing the background fluorescence.

Although IVM has a long history, the number of studies using IVM started to increase only after the new millennium,^3^ largely due to development of the 2P excitation microscope. The process of 2P absorption is a nonlinear optical one that depends on the square of the light intensity. Taking advantage of this property, Denk *et al*. invented 2P excitation microscopy (2PEM),^4^ which is now widely used across the broad field of life science. The first advantage of 2PEM is that it uses near‐infrared wave length for excitation (700–1300 nm). The primary obstacle that prevents us from seeing deep tissues is light scattering – more specifically, Rayleigh scattering. The degree of Rayleigh scattering is inversely correlated with the fourth power of the wavelength. Therefore, near‐infrared light can penetrate the tissues more deeply than visible light. Furthermore, the absorption of hemoglobin, one of the major light‐absorbing molecules, is markedly lower in the near‐infrared range than the visible light range^5^ (Fig. 1a). The second advantage of 2PEM is related to the nonlinear optical process. In conventional single‐photon excitation, all fluorescent molecules along the light path emanate fluorescence. Therefore, to obtain a clear image of the focal plane, the fluorescence light derived from out‐of‐focus planes must be excluded, such as by using a confocal pinhole. This process also reduces the signal from the focal plane. In contrast, only the molecules at the focal plane are excited in 2PEM (Fig. 1b), enabling maximum recovery of photons from the molecules of interest. Through these principles, 2PEM now allows us to see brain tissue to more than 1 mm depth, in contrast to traditional microscopy, which can visualize only structures closer to the surface of tissues.^6^

### Fluorescence labeling for 2PEM

Much like conventional fluorescence microscopy, 2PEM also requires tissues to be labeled with fluorescent molecules, except when observing autofluorescent molecules or using second harmonic generation. Fluorescent dyes have been routinely used for the staining of tissue sections or tissue culture cells; however, staining of cells or molecules of interest in live animals is not an easy task, limiting the application of fluorescent dyes in IVM. Exceptions are the vasculature, which can be labeled by intravenous injection of fluorescent dyes,^7^ and immune cells, which can be labeled *ex vivo* and introduced back into the host.^8^ Meanwhile, there are two advantages of fluorescence proteins, which derive from their being “genetically encoded” dyes. First, the fluorescent proteins will be produced as long as the cells carry the fluorescent protein genes. After the establishment of transgenic mice expressing the fluorescent proteins, there is no need to add anything for the visualization. Thus, fluorescence proteins are a very stable and inexpensive labeling technique. The second advantage is that the cells of interest can be labeled with fluorescent proteins expressed from a cell type‐specific promoter/enhancer. For example, lymphocyte‐specific expression of fluorescent proteins has visualized how immune cells communicate with each other in time‐ and space‐specific manners.^9^ In other studies, cells are labeled with fluorescent proteins by ubiquitously expressed promoters and distinguished by the characteristic morphology of each cell type^10^, ^11^ Markers for the cell membrane and nucleus are often used for this purpose.^12^, ^13^, ^14^ Notably, a number of mouse reporter lines have been developed by Aizawa and his colleagues, who utilized the ROSA26 locus for ubiquitous expression *in vivo*,^12^, ^14^ providing the basis for IVM by 2P excitation microscopes. In addition, we recently established mouse lines expressing a morphology marker named NuCyM, which enables us to distinguish the nucleus, plasma membrane and cytoplasm by 2PEM.^15^ With NuCyM, images acquired by 2PEM can be converted to H&E‐like images.

### Biosensors based on the principle of Förster resonance energy transfer

**Figure 2:**
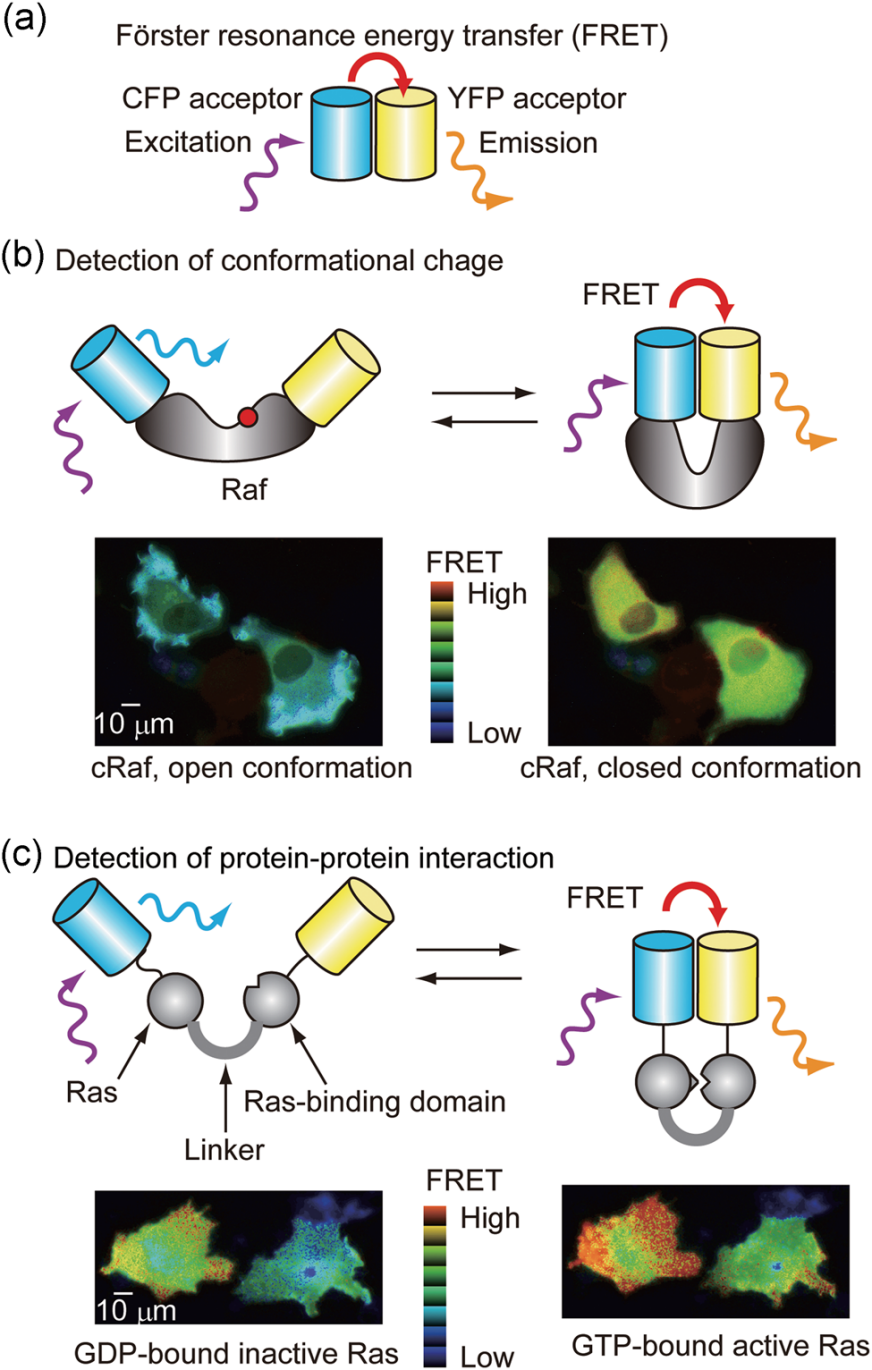
Typical examples of the Förster resonance energy transfer (FRET) biosensor. (**a**) FRET is a process by which a donor fluorophore in an excited state non‐radiatively transfers its energy to a neighboring acceptor fluorophore. (**b**) Many protein kinases including Raf adopt an open active conformation and closed inactive conformation. The former is often induced by phosphorylation as indicated by the red circle. By fusing the donor and acceptor fluorophore, this conformational change can be detected by the efficiency of FRET. In each pixel of these FRET images, the color hue reflects the fluorescence intensity ratio of acceptor versus donor in eight grades, and the intensity of each color reflects the concentration of the probe. (**c**) In another FRET biosensor design, signal‐induced protein‐protein interaction can be detected by FRET. In this example, Ras and the Ras‐binding domain of Raf are tandemly connected, and further sandwiched by the donor and acceptor fluorophores. Upon Ras activation, the guanosine triphosphate‐bound active Ras binds to Raf and thereby provokes FRET.

The utility of fluorescent proteins has been further extended by the development of a variety of biosensors. There are a plethora of genetically encoded probes;^16^, ^17^ however, here we will only consider biosensors based on the principle of Förster resonance energy transfer (FRET), because we will refer to transgenic mice expressing the FRET biosensors in the following sections. FRET is a process by which a donor fluorophore in an excited state non‐radiatively transfers its energy to a neighboring acceptor fluorophore.^18^, ^19^ Most of the currently available FRET biosensors use cyan fluorescent protein (CFP) and yellow fluorescent protein (YFP) as the donor and acceptor, respectively (Fig. 2a). By fusing the donor and acceptor proteins to the protein of interest, the activity change of the protein, which is caused by the conformational change, can be monitored by the change in FRET efficiency. As a typical example, we adduce a FRET biosensor for cRaf (Fig. 2b). cRaf adopts open active and closed inactive conformations in the manner of many protein kinases. By fusing CFP and YFP to both ends, a FRET biosensor for cRaf was generated, in which FRET efficiency was inversely correlated with the activity of cRaf.^20^ Alternatively, protein–protein interaction, which is a key step in signal transduction, can be monitored by measuring the FRET efficiency (Fig. 2c). For example, Ras is known to bind to cRaf in an activation‐dependent manner. By fusing Ras and the Ras‐binding domain of cRaf, a FRET biosensor for Ras was generated, in which FRET efficiency was correlated with the activity of Ras. Upon growth factor stimulation, guanosine diphosphate (GDP)‐bound inactive Ras becomes guanosine triphosphate (GTP)‐bound active Ras and transmits a signal to cRaf. By the use of the probe, it was found that this GDP‐GTP exchange reaction occurs primarily at the free‐edge of epithelial cells, solving the enigma known as contact inhibition of cell growth.^21^

## EXPERIMENTAL PATHOLOGY BY IVM WITH GENETICALLY ENCODED BIOSENSORS

Currently, the field of neuroscience has seen by far the most applications of IVM with 2PEM. Because the refractive index of the brain is relatively homogenous, light scattering occurs much less in the brain than in the other organs, allowing deeper penetration of excitation light, up to 1 to 2 mm from the brain surface. Moreover, a glass window implanted to the skull allows researchers to observe neurons without motion artifacts and to revisit the same neuron for several months. Because there are a huge number of articles on the intravital imaging of mouse brains, here we will refer only to several excellent review papers,^22^, ^23^, ^24^ and proceed to IVM in the other tissues.

What are the events that we can see by IVM but not by conventional microscopy? A good example is cell migration, such as that in leukocyte extravasation or epithelial cell wound healing. We know that leukocytes extravasate from the blood vessels and we may imagine how they seek bacteria or diseased cells, but without seeing live images, we can hardly predict with any precision how cells move around in the tissues. Other examples are those events which fluctuate with time. For example, endothelial cell damage induces both pro‐ and anti‐thrombotic signaling cascades, leading to the cycles of thrombus formation and resolution. This phenomenon cannot be foreseen by static images. Similarly, temporal changes of signaling molecule activities are also hard to predict by conventional immunohistochemical techniques. Below, we will consider some key applications of IVM in detail.

### Visualization of neutrophil extravasation

Next to neuroscience, immunology is the second largest research area capitalizing on the advantages of IVM.^9^, ^25^ This is probably because a period of several hours of observation under anesthesia is sufficient to study the dynamic interaction between immune cells and target cells. Two‐photon microscopy analysis of lymphocytes has already been reviewed.^26^ Here we focus on the migration of neutrophils.

**Figure 3:**
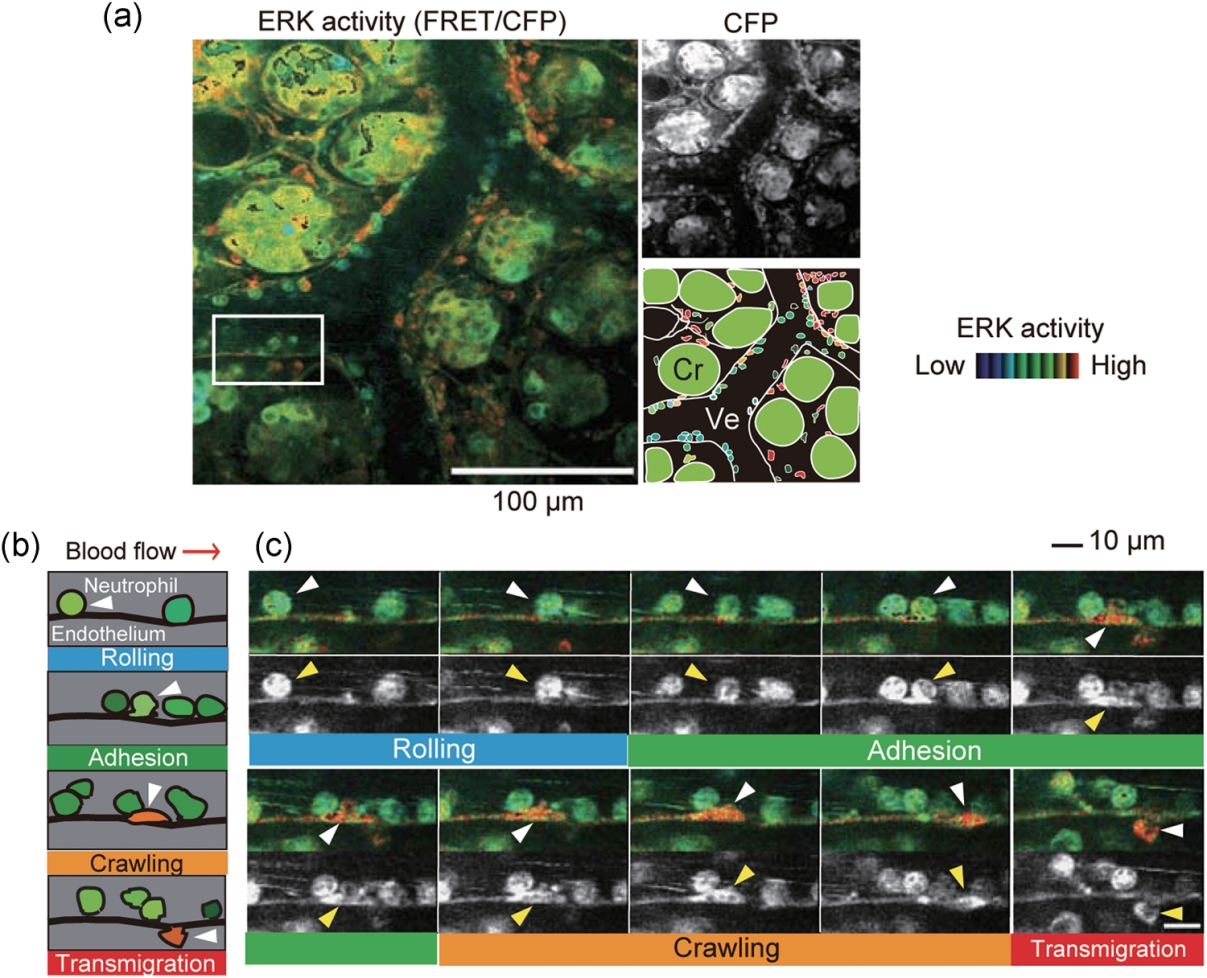
An example of intravital imaging of molecular activity during neutrophil recruitment to the lamina propria of the intestinal mucosa. (**a**) A representative Förster resonance energy transfer (FRET)/cyan fluorescent protein (CFP) image of the inflamed intestine of a mouse expressing the FRET biosensor for extracellular signal‐regulated kinase (ERK). Images are shown in intensity‐modulated display (IMD) mode using eight ratios with 32 intensities and a CFP image in grayscale, with a schematic view of this region. Cr, crypt; Ve, venule. (**b**) Schematic of the four steps of extravasation. White arrowheads indicate the same neutrophil at different time points. (**c**) Time‐lapse FRET/CFP and CFP images of neutrophil extravasation. The boxed region (**a**) was magnified and shown in a time series. Reproduced from Mizuno *et al*.^31^

Extravasation of neutrophils is the hallmark of acute inflammation. IVM has revealed that the neutrophil recruitment involves four steps: rolling, adhesion, crawling and transmigration.^27^, ^28^, ^29^ It has been shown *in vitro*, but not *in vivo*, that various intracellular signaling molecules are activated in neutrophils and endothelial cells during extravasation of neutrophils. We previously developed transgenic mice expressing FRET biosensors for protein kinase A (PKA) and extracellular signal‐regulated kinase (ERK).^30^ By observing the inflamed intestines of these transgenic mice by 2PEM, we visualized the activity change of PKA and ERK in neutrophils.^31^, ^32^ First, neutrophils roll on and adhere firmly to the endothelial cells (Fig. 3). Neutrophils then crawl for a few minutes, and transmigrate to the interstitial tissue. ERK is activated during the adhesion process, supporting the positive role of ERK in the extravasation of neutrophils. Pharmacological perturbation experiments have shown that this ERK activation is dependent on leukotriene B (LTB). Meanwhile, it was found that prostaglandin E2 (PGE_2_) activates PKA and suppresses ERK. Thus, LTB_4_ recruits neutrophils to the inflamed lesion by ERK activation, and PGE_2_ stops neutrophils from functioning as scavengers of damaged tissues by PKA‐mediated ERK inactivation, highlighting the antagonistic action of these two chemical mediators derived from arachidonic acid.

Let us consider one other example of a secret of life that was uncovered by IVM. For many years, people believed that neutrophils recruited to the inflamed tissue live only for a short period of time, long enough to conduct their missions as tissue destroyers and scavengers, and then are doomed to die. This dogma has now been challenged based on observations made using IVM. It is reported that, upon sterile injury of the liver, the recruited neutrophils create new vasculature, through which they return back to the blood.^33^, ^34^ Under conditions of ischemia‐reperfusion as well, neutrophils return to the vasculature^35^ as do the lymphocytes. These phenomena, called either reverse migration or disrupted transendothelial migration, contribute to systemic inflammation, particularly in the lung.^36^

### Dynamics of thrombosis

Formation of stable thrombus has been visualized with conventional wide‐field microscopy or confocal microscopy,^37^, ^38^, ^39^, ^40^ and high‐resolution imaging has further revealed that the platelet aggregation could be divided into two phases.^41^, ^42^, ^43^ First, the discoid platelets are tethered loosely to the blood vessels. Second, the platelets change their shape to form a core of fully activated platelets. Meanwhile, *in vitro* biochemical studies have delineated the intracellular signal transduction cascades that promote or inhibit thrombus formation.^44^ One of the key accelerators of thrombus formation is ERK, which mediates adenosine diphosphate‐induced TxA_2_ generation and activation of integrin α_IIb_β_3_.^45^, ^46^ On the other hand, PKA is the major brake of thrombus formation.^44^, ^47^, ^48^, ^49^, ^50^ Both of the major anti‐thrombotic molecules, prostacyclin I_2_ and nitric oxide, are known to suppress thrombus formation via PKA activation.^48^, ^51^ However, spatio‐temporal regulation of ERK or PKA during thrombus formation has not been observed due to technical difficulties. By using transgenic mice expressing the FRET biosensor for ERK or PKA, Hiratsuka *et al*. visualized the activity change of ERK and PKA during thrombus formation in platelets.^52^ As anticipated, ERK is activated rapidly at the core of contracting platelet aggregates. However, unexpectedly it was found that PKA is also activated almost simultaneously with ERK. Thus, by stimulating both pro‐ and anti‐thrombotic signaling molecules, fine tuning of the thrombus formation could be accomplished. In fact, the thrombus formation is not a straightforward process, but is the outcome of repeated growth and resolution phases. This is another good example of the visualization of signaling activity in live tissues helping us to understand pathogenesis in the real world.

### Wound healing of epithelial cells

**Figure 4:**
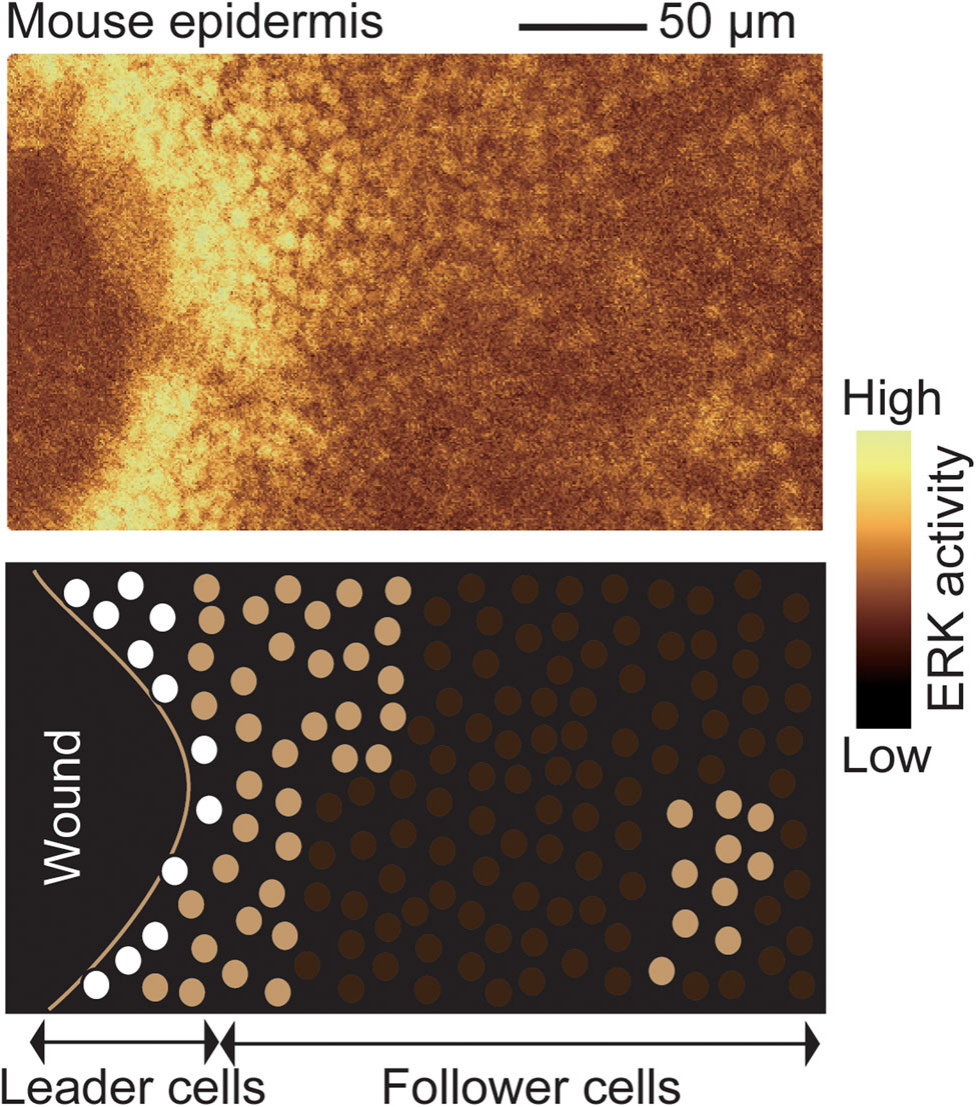
Wound healing and propagation of extracellular signal regulated kinase (ERK) activation. The epidermis of a transgenic mouse expressing a Förster resonance energy transfer (FRET) biosensor for ERK was subjected to epithelial wounding 12 h before imaging. The FRET/cyan fluorescent protein (CFP) ratio is shown by gold pseudocolors (top panel). The cartoon schematically shows the ERK activation. The white circles indicate nuclei of the leader cells showing highest ERK activity, whereas gray circles indicate nuclei of follower cells consisting of ERK activation waves.

In many textbooks of basic pathology, wound healing is described as a typical example of a tissue repair process, wherein inflammation, cell migration, cell proliferation and tissue remodeling occur in an organized fashion.^53^ Meanwhile, in cell biology, wound healing has been studied extensively as a representative form of collective cell migration of epithelial cells, wherein a number of mechanisms, such as chemotaxis, haptotaxis, durotaxis, and so on, underlie the organized cell movement.^54^ One of the key questions in regard to collective cell migration is how the moving cue is propagated from the leader cells to the follower cells.^55^ In previous studies, we noticed that the activation of epidermal growth factor receptors (EGFRs) and their canonical downstream signaling protein ERK could be propagated between epithelial cells both in tissue culture cells and in the epidermis of living mice.^56^, ^57^ Interestingly, big waves of ERK activation are generated at the wound edge of the epidermis and propagated to the follower cells up to several millimeters distance^56^, ^58^ (Fig. 4). Pharmacological inhibition of A distintegrin and metalloprotease (ADAM), EGFR or ERK, perturbed the ERK activation wave and also collective cell migration during epidermal wound healing.^56^ ADAM is required for shedding of the epidermal growth factor (EGF)‐family protein; collectively, therefore, these observations clearly indicate that the ERK activation wave is propagated by activation of the EGF‐signaling cascade and that the ERK activation wave drives the follower cells to go forward to chase the leading cells and to efficiently fill the defect.

Is such an ERK activation wave observed in other tissues? We noticed a similar phenomenon within intestinal epithelial cells, but its role in migration has not been addressed.^59^ What about urothelium? Urothelial cells exhibit ERK activation upon stretching;^60^ however, no ERK activation wave is observed at the wound.^61^ Unexpectedly, a critical difference between the epidermis and urothelium was revealed by 2PEM. The urothelium, but not the epidermis, can glide over the interstitial tissues, which appears to be the reason why an ERK activation wave is not required for the wound healing of urothelium.

## CANCER RESEARCH BY IVM AND GENETICALLY ENCODED FLUORESCENT BIOSENSORS

For many years, it has been known that cancer tissues consist of heterogeneous cell populations. Cancer cells themselves are heterogeneous both in phenotypes and genotypes. Furthermore, growing cancer cells always recruit vascular cells, interstitial cells and immune cells. Microscopy is the best approach to study the heterogeneity of cancer tissues, and, therefore, the gold standard for the diagnosis of cancer. IVM further provides great opportunities to decipher the dynamic interaction among cancer cells, interstitial cells, immune cells, vascular cells, and so on. There are several excellent review papers on the contribution of IVM to the understanding of cancer cell intravasation, metastasis and sensitivity to anticancer drugs.^62^, ^63^, ^64^, ^65^, ^66^ Here we focus on cancer research by IVM with genetically encoded fluorescent biosensors. In the pre‐green fluorescent protein (GFP) era, IVM in cancer research was mostly focused on the visualization of tumor vasculature.^62^ GFP technology enabled researchers to observe live tumor cells for a long period sufficient to analyze the mode of invasion.^67^ Introduction of 2PEM further accelerated IVM of tumor tissues because of its superiority for deep tissue imaging.^68^ Moreover, the development of peripheral devices such as the imaging window has created ideal conditions for observing the pathophysiology of tumor environments by 2PEM.^69^, ^70^ As already stated, one advantage of GFP is that it can be used not only to label tumor cells, but also to supply a variety of biosensors that monitor the function of cancer cells and the interaction between cancer cells and host cells. For example, by using a FRET biosensor for the stress‐responsive kinase Tak1, it has been shown that the Tak1 activity is high at the periphery of tumors, suggesting that tumor cells at the periphery of the tumor mass are exposed to higher levels of stress than those in the central region of the tumor mass.^71^ Below, we will consider additional examples of the benefit of genetically encoded fluorescent biosensors used in combination with IVM.

### Regulation of glioblastoma invasion by Rho‐family GTPases

**Figure 5:**
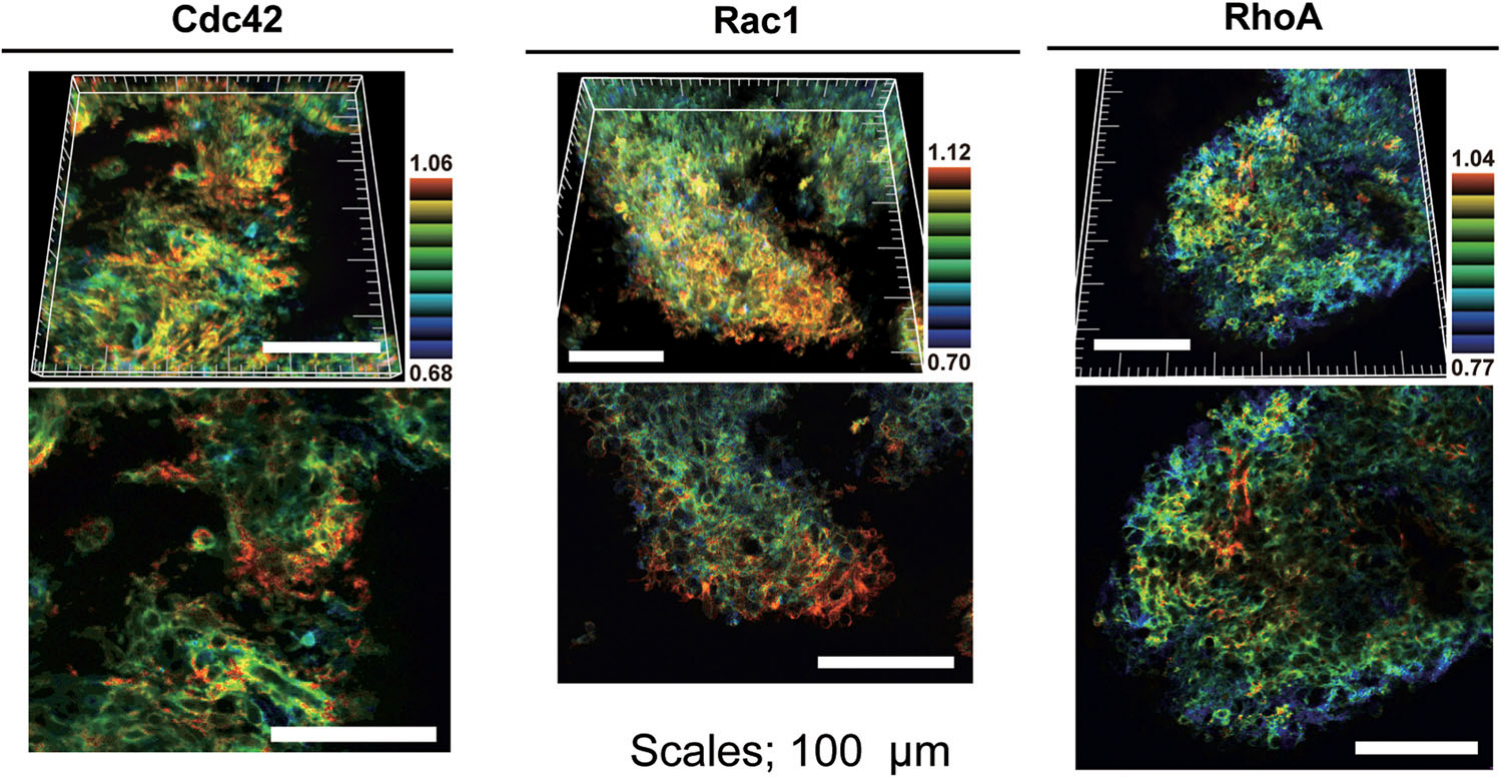
Activity of Rho‐family GTPases in glioblastoma invading into the brain parenchyma. C6 glioblastoma cells stably expressing Förster resonance energy transfer (FRET) biosensors for Cdc42, Rac1 and RhoA were injected into rat brain. After 7 days, the invasion front of the tumor was imaged under a two‐photon excitation microscope. The three‐dimensional reconstructed images are shown in intensity‐modulated display (IMD) mode with 32‐intensity in eight‐ratio. Reproduced from Hirata E *et al*.^73^

As stated earlier, brain tissue has a great advantage for use in intravital imaging because the skull can be firmly stabilized with a fixing device. By 2PEM, it has been visualized that glioblastoma cells move much faster in the perivascular space than in the brain parenchyma in mouse or rat glioblastoma models.^72^, ^73^ Cell migration is generally regulated by Rho‐family GTPases, which dictate actin polymerization and actomyosin contraction.^74^, ^75^, ^76^ Hirata *et al*. studied the role of Rho‐family GTPases in glioblastoma invasion by using FRET biosensors for Rho‐family GTPases (Fig. 5).^73^ Rac1 and Cdc42, which promote actin polymerization, are activated in glioblastoma cells invading into the brain parenchyma. Meanwhile, RhoA, which induces actomyosin contraction, is activated primarily in the center of tumor tissue, particularly at the perivascular region, suggesting that the balance of GTPase activities may control the migratory property of glioblastoma cells. How, then, is such heterogeneity of GTPase activity generated in growing glioblastoma cells? Time‐lapse imaging of Rac1 activity over several days has revealed that Rac1 activity in glioblastoma cells fluctuates over a timescale, substantially longer than that of the replication cycle.^77^ When glioblastoma cells were embedded in gel, the high Rac1 activity cell population was found to invade into the gels, leading the other cells.^73^ RNA‐Seq analysis of Rac1‐hi and Rac1‐lo cells revealed a signaling network that comprises both positive and negative feedback loops, which may be sufficient to cause oscillation of the activity change of Rho‐family GTPases. Such an intrinsic mechanism of generating heterogeneity may be beneficial to adapt to variations in the tissue environment.

### Stem cell property of breast cancer

Transgenic mice expressing fluorescent proteins or genetically encoded fluorescent biosensors are also frequently used to improve our understanding of naturally occurring cancers. For example, the roles of oncogene and tumor suppressor genes in oncogenesis have been extensively studied in a variety of transgenic mice, in which fluorescent proteins are expressed in the cell types of interest.^78^ We crossed transgenic mice expressing a FRET biosensor for ERK with mouse mammary tumor virus (MMTV)‐Neu transgenic mice, which are widely used as a mouse model of HER2/Neu‐positive luminal‐type breast cancer.^79^ By 2PEM, significant heterogeneity of ERK activity was observed among the breast cancer cells. Intriguingly, the ERK activity was inversely correlated with the efficiency of tumor sphere generation *in vitro* and the expression of the cancer stem cell markers CD49f, CD24 and CD61, suggesting that ERK negatively regulates the stem cell property. Meanwhile, the role of ERK activity in the prognosis of human breast cancer patients is controversial. One report claimed that high ERK activity in breast cancer tissues predicts poor prognosis of patients,^80^ whereas others reports proposed that high ERK activity in breast cancer tissues is associated with improved survival.^81^, ^82^, ^83^ ERK is a major signaling output of receptor‐type tyrosine kinases including HER2/Neu/ErbB2. Thus, our observation in mice suggests that high ERK activity accelerates tumor growth and at the same time suppresses the stemness of cancer cells. It is likely that the ERK activity varies significantly among the different tumor regions, which renders the correlation of ERK activity to prognosis controversial.

### Enhanced permeability of tumor vessels

**Figure 6:**
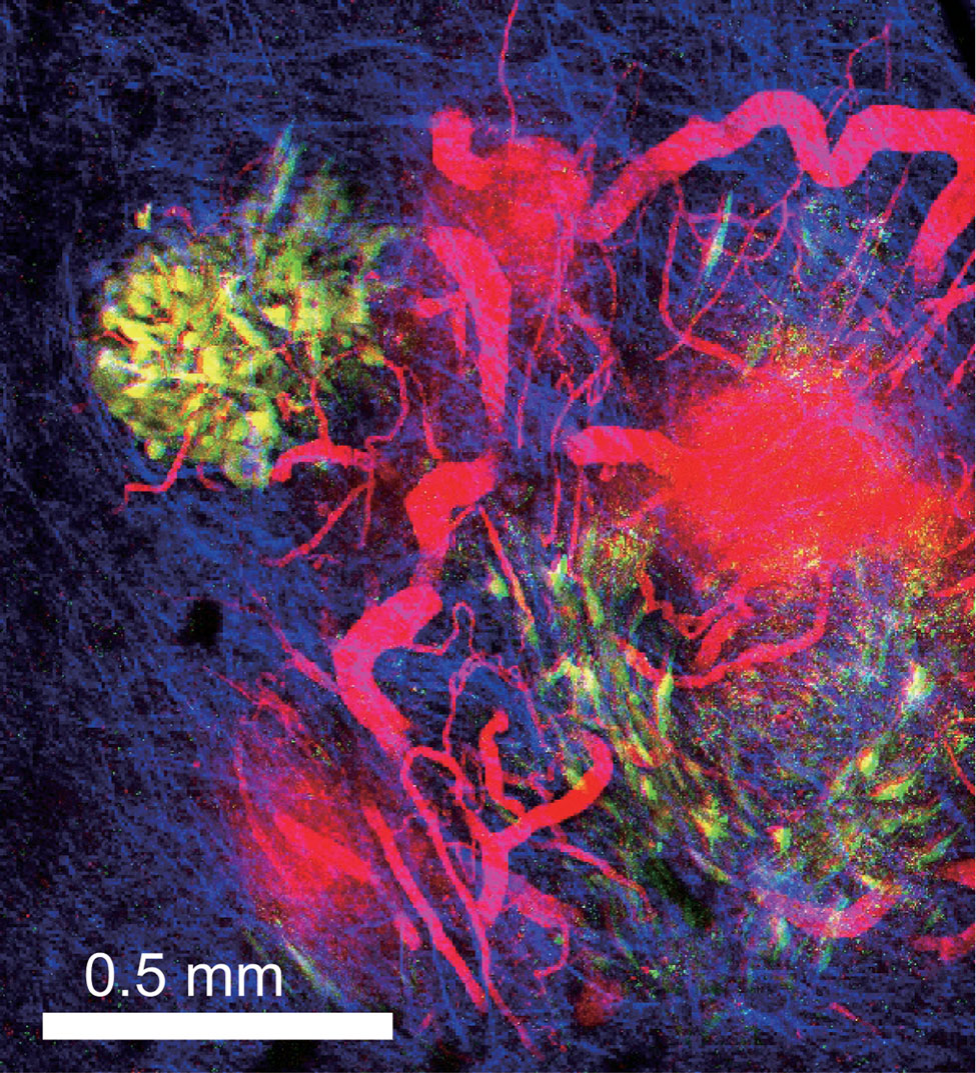
Increased vascular permeability. Panc‐02 cancer cells (yellow) implanted subcutaneously into a C57BL/6N Jcl mouse were observed by two‐photon excitation microscopy (2PM). Texas red‐conjugated dextran was injected to visualize blood vessels (red). Collagen fibers were visualized by second harmonic generation (blue).

IVM is particularly useful to visualize the dynamic properties of cancer tissues, such as the enhanced permeability and retention (EPR) effect. EPR is a hallmark of cancer tissue and is closely related to angiogenesis and metastasis^84^, ^85^, ^86^ (Fig. 6). Harney *et al*. have proposed the presence of a tumor microenvironment of metastasis, where transient vascular permeability and tumor cell intravasation simultaneously occur.^87^ Vascular endothelial growth factor (VEGF) is known as the key molecule controlling the vascular hyperpermeability. Within cells, many intracellular signaling molecules, such as Src‐family tyrosine kinases, PI 3‐kinases and phospholipase C, have been shown to mediate the actions of VEGF.^88^ However, signaling cascades that may compete with the VEGF signaling cascade have not received much attention in cancer research. For example, the cAMP signaling cascade was shown to maintain normal endothelial barrier function,^89^, ^90^, ^91^ but its role in the vascular hyperpermeability in tumor tissue remained elusive. Using a combination of FRET biosensors and 2PEM, a more recent study revealed that the activity of PKA is significantly lower in tumor endothelial cells than in normal endothelial cells.^92^ Further experiments showed that VEGF decreased PKA activity in normal endothelial cells, but that a VEGF inhibitor, Motesanib, increased PKA activity in tumor blood vessels, indicating that the decreased PKA activity causes vascular hyperpermeability. Importantly, in human umbilical vascular endothelial cells (HUVECs), which are the most widely used endothelial cells for *in vitro* experiments, VEGF does not decrease, but rather increases PKA activity,^93^ highlighting the critical difference between *in vivo* and *in vitro* conditions. Since the EPR is often utilized to deliver anticancer drugs, any knowledge pertaining to control of the permeability of the tumor vasculature would be of great clinical significance.^94^

### Anticancer drug actions

Recent developments in IVM that have shed light on the actions of anticancer drugs have been extensively reviewed already.^64^ Here, we refer to the mechanisms underlying the remittance after anticancer drug treatment. Genetic heterogeneity in cancer cell populations is of course one of the primary reasons for such remittance, but it has also been shown that the microenvironment of cancer cells is a critical factor for the drug response of cancer cells.^95^ Using 2PEM and a FRET biosensor for ERK, Hirata *et al*. showed that a BRAF inhibitor activates ERK in melanoma‐associated fibroblasts by a mechanism known as ‘paradoxical activation’, and that the activated fibroblasts provide a “safe haven” for melanoma cells to tolerate BRAF inhibition.^96^ This and other studies have highlighted the importance of the activated fibroblasts, as described in a recent review.^97^

The development of an effective anticancer drug for inoperable pancreatic cancer patients is urgently needed. Combination therapy with gemcitabine and nab‐paclitaxel is the current gold standard, but the overall survival is still less than 1 year.^98^ By the use of a FRET biosensor for RhoA, Timpson and colleagues showed that RhoA activation at the invading front of the pancreatic cancer is critical for the invading capacity of pancreatic cancer.^99^ They further examined the role of the principal effector of RhoA, ROCK, on the sensitivity to gemcitabine and nab‐paclitaxel. Using a FRET biosensor for cyclin‐dependent kinase 1 (Cdk1) as a surrogate marker of M‐phase cell cycle arrest induced by gemcitabine/nab‐paclitaxel, they found that the ROCK inhibitor Fasudil can improve the effect of the combination therapy in patient‐derived xenografts.^100^ Because the RhoA signaling cascade is activated in pancreatic cancer,^101^ inhibitors of the RhoA signaling cascade may provide a promising molecular target for anticancer drugs. Another potential target molecule is Src, which is highly expressed in pancreatic cancer.^102^, ^103^ However, the clinical trials of anti‐Src drugs have failed to show effectiveness in combination with gemcitabine in advanced pancreatic cancer.^104^, ^105^ This may be due to the poor delivery of the Src inhibitors. In fact, using a FRET biosensor for Src, it has been shown that the pancreatic cells distal to the vasculature respond poorly to the Src inhibitor dasatinib.^106^ Thus, the genetically encoded biosensors could help to reveal the discrepancy between the *in vitro* and *in vivo* observations.

## FUTURE PROSPECTIVES

The recent success of IVM does not come from a single invention. Indeed, the handling of very large 4D images has become possible only with the advent of information technology. Introduction of adaptive optics, software to alleviate motion artefacts during imaging,^107^, ^108^ and instruments for organ fixation have also improved the quality of images. Moreover, the introduction of artificial intelligence will greatly help in analyzing the vast amounts of imaging data generated by IVM. Thus, IVM will continue to expand its role in experimental pathology. However, what should be emphasized here is the continued importance of investigation by the human eye. Artificial intelligence (AI) may be better at answering labor‐intensive questions than humans, but AI cannot answer unasked questions. The questions hidden in the images can be mined only by scientists with profound knowledge of the histology. IVM will provide unlimited opportunities to identify clues to understand the pathogenesis and the keys to developing new remedies.

## DISCLOSURE STATEMENT

None declared.

## AUTHOR CONTRIBUTIONS

Drafting the manuscript and figures, MM and KT.
